# Comparing navigated transcranial magnetic stimulation mapping and “gold standard” direct cortical stimulation mapping in neurosurgery: a systematic review

**DOI:** 10.1007/s10143-020-01397-x

**Published:** 2020-10-03

**Authors:** Hanne-Rinck Jeltema, Ann-Katrin Ohlerth, Aranka de Wit, Michiel Wagemakers, Adrià Rofes, Roelien Bastiaanse, Gea Drost

**Affiliations:** 1 Department of Neurosurgery, University Medical Center Groningen, Hanzeplein 1, P.O. Box 30.001, 9700 RB Groningen, the Netherlands; 2 Center for Language and Cognition Groningen, University of Groningen, Oude Kijk in ’t Jatstraat 26, 9712 EK Groningen, the Netherlands; 3 Faculty of Medical Sciences, University of Groningen, Antonius Deusinglaan 1, 9713 AV Groningen, the Netherlands; 4 Center for Language and Brain, National Research University, Higher School of Economics, Moscow, Russian Federation

**Keywords:** nTMS, Navigated transcranial magnetic stimulation, Direct cortical stimulation, DCS, Mapping

## Abstract

The objective of this systematic review is to create an overview of the literature on the comparison of navigated transcranial magnetic stimulation (nTMS) as a mapping tool to the current gold standard, which is (intraoperative) direct cortical stimulation (DCS) mapping. A search in the databases of PubMed, EMBASE, and Web of Science was performed. The Preferred Reporting Items for Systematic Reviews and Meta-Analyses (PRISMA) guidelines and recommendations were used. Thirty-five publications were included in the review, describing a total of 552 patients. All studies concerned either mapping of motor or language function. No comparative data for nTMS and DCS for other neurological functions were found. For motor mapping, the distances between the cortical representation of the different muscle groups identified by nTMS and DCS varied between 2 and 16 mm. Regarding mapping of language function, solely an object naming task was performed in the comparative studies on nTMS and DCS. Sensitivity and specificity ranged from 10 to 100% and 13.3–98%, respectively, when nTMS language mapping was compared with DCS mapping. The positive predictive value (PPV) and negative predictive value (NPV) ranged from 17 to 75% and 57–100% respectively. The available evidence for nTMS as a mapping modality for motor and language function is discussed.

**Electronic supplementary material:**

The online version of this article (10.1007/s10143-020-01397-x) contains supplementary material, which is available to authorized users.

## Introduction

In neurosurgical practice, often lesions are encountered, which are invading into eloquent brain regions or have a close relation with eloquent brain structures (i.e., motor, language, or other cognitive function). This is true for glioma surgery, for different kinds of vascular surgery, epilepsy surgery, and for surgical procedures aiming at resection of lesions like metastasis, meningiomas, and cavernomas. To maximize the safety and extent of resection of neurosurgical procedures, multiple mapping and monitoring modalities have been developed. The review of Ottenhausen et al. [37] gives a good overview of the available techniques. Besides nTMS, magnetoencephalography (MEG), diffusion tensor imaging-fiber tracking (DTI-FT), and functional magnetic resonance imaging (fMRI) are available for preoperative mapping. In the intraoperative setting, DCS mapping and monitoring and subcortical stimulation (SCS) mapping are useful techniques. MEG records neuronal activity by measuring magnetic fields produced by electric currents in the brain. With the MEG technique, spatiotemporal mapping of motor, somatosensory, language, auditory, and visual functions can be performed [37]. The high cost of MEG, and therefore the limited availability, is a big disadvantage of this technique. DTI-FT enables subcortical white matter fiber tract delineation. However, it is a purely radiologic anatomical imaging technique that does not include physiological functional data [37]. Traditionally, fMRI is the most available and employed preoperative mapping technique for localization of eloquent cortical brain areas for different types of neurological functions. One of the problems of the fMRI technique is that blood-oxygen-level-dependent (BOLD) imaging shows blood oxygenation level as a surrogate parameter of neuronal activity. It cannot be discerned if a BOLD signal represents a critical cortical area or only a participatory, non-essential cortical area for the tested neurological function. The performance of fMRI is suboptimal in the vicinity of brain tumors [10]. Regarding sensitivity and specificity of fMRI motor function localization, the literature is not unambiguous [37]. Also, fMRI language localization sensitivity and specificity show a very broad range, limiting its ability as a presurgical mapping tool [13]. In general, only fMRI mapping in adjunct to other methods is advised [37].

In recent years, many studies on the use of navigated transcranial magnetic stimulation (nTMS) as a preoperative mapping tool have been published. nTMS enables inhibition or excitation of a cortical area by way of repetitive or single transcranial magnetic pulses, respectively. This technique is appealing, because it can be conducted preoperatively in a controlled environment. The procedure can be repeated as often as is deemed necessary by the treating physician. This in contrary to intraoperative direct cortical stimulation (DCS) mapping, where fatigue and loss of optimal concentration during awake procedures, epileptic seizures as well as duration of the surgery can be limiting factors for the mapping procedure. Furthermore, the cortical surface that can be mapped intraoperatively is limited to the extent of exposure of brain cortex by the craniotomy. Nonetheless, intraoperative mapping is still considered the gold standard amongst most neurosurgeons.

The aim of this review is to give a contemporary overview of the existing literature comparing nTMS mapping to DCS mapping techniques. In the previous literature, a smaller scale review from Takahashi et al. [53] on localization of motor function by way of nTMS appeared in 2013, in which 11 articles were included. The recent review and meta-analysis of Raffa et al. [46] from 2019 also focuses solely on motor mapping, with an emphasis on the effect on oncologic treatment outcome, and is not informative concerning the comparison of nTMS and DCS mapping. In this publication, the authors found a significantly reduced risk of postoperative new permanent motor deficit and an increased rate of gross-total resection (GTR), in favor of the use of nTMS. Also, a smaller craniotomy size and a trend toward a reduction in the duration of the surgery were found in this meta-analysis. However, the authors also conclude that there is a need for high-level evidence from multicenter randomized controlled studies. Our review adds to the abovementioned literature by considering not only motor mapping but also language mapping. For other neurological functions, no comparative data could be found at this moment.

## Materials and methods

The primary research objective was formulated as follows: the comparison of mapping techniques in patients with a neurosurgical intervention in an eloquent brain area (population) undergoing preoperative cortical mapping using nTMS and cortical mapping by DCS for neurological function localization (outcome) in prospective and retrospective comparative case series and cohort studies (study design).

### Search strategy

On 17 September 2019, a literature search was performed in the electronic databases of PubMed, EMBASE, and Web of Science. A search strategy was formulated for each of the three databases (see Table 1 for the PubMed search strategy; the comparable search strings for all three databases are given in Supplement 1). The review was conducted according to the PRISMA guidelines and recommendations.

**Table 1:** Search strategy as applied in the electronic database of PubMed

((navigated transcranial magnetic stimulation OR navigated TMS) **OR** ((“Transcranial Magnetic Stimulation”[Mesh] OR transcranial magnetic stimulation*[tiab] OR TMS[tiab] OR rTMS[tiab]) **AND** (((intraoperat*[tiab] OR intra-operat*[tiab] OR during surg*[tiab] OR (awake[tiab] AND surgery[tiab]) OR intracranial[tiab]) *AND* (mapping[tiab] OR cortical stimulat*[tiab] OR subcortical stimulat*[tiab])) OR direct cortical stimulat*[tiab] OR direct electrical stimulat*[tiab] OR cortical stimulation mapping[tiab] OR intraoperative stimulat*[tiab] OR intra-operative stimulat*[tiab] OR direct stimulation[tiab] OR ((direct[tiab] NOT (“direct current”[tiab] OR tdcs[tiab])) *AND* (cortical stimulat*[tiab] OR electrical stimulat*[tiab])) OR dcs[tiab]))) **NOT** (“Animals”[Mesh] NOT “Humans”[Mesh])	

### Study selection criteria

**Fig. 1:**
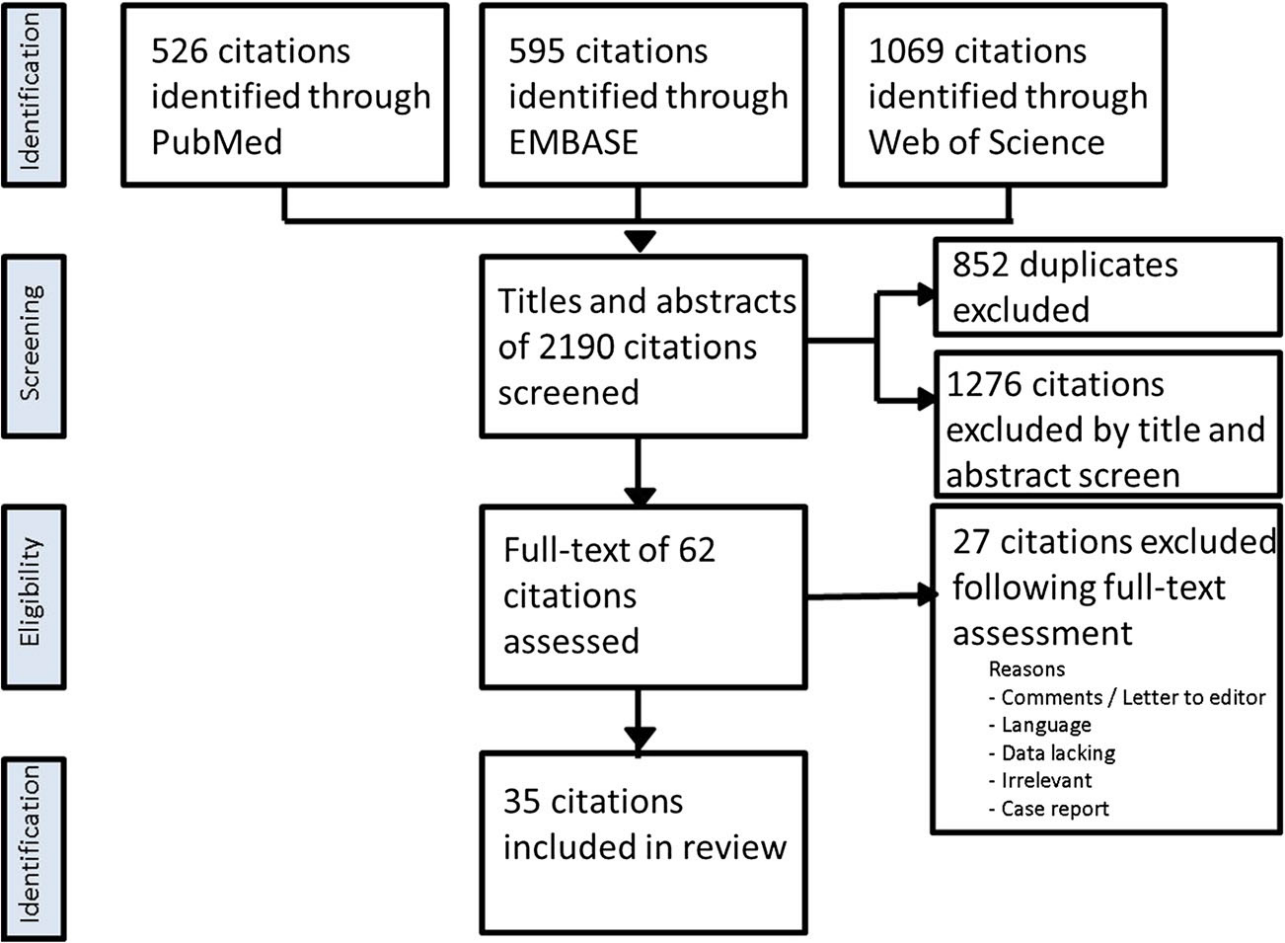
PRISMA flowchart of the selection process of articles included in the review

The steps of selection of the articles for inclusion are shown in the flowchart (Fig. 1). Articles that met the following criteria were included: (1) articles describing patients undergoing both a nTMS mapping procedure and a DCS mapping procedure. (2) A comparison in function localization between both mapping techniques was performed. The study selection procedure was performed by three of the authors (H-RJ, A-KO, AW). Each citation was checked by at least two different researchers. Disagreement was resolved by discussion. The following languages were allowed for inclusion: English, German, French, Italian, and Spanish. There was no restriction in the type of neurological function that was being mapped, although motor and language were the predominant neurological functions investigated in most publications. The references of the articles selected for full-text reading were hand searched for new eligible citations. This did not lead to the addition of any new citations. Comments, letters to the editor, and author replies were excluded because they contained no new experimental data. Case reports describing a single patient were excluded in this review.

### Quality assessment of included articles

The study design of the included articles in this review was noted. Furthermore, the articles were scored on four different domains as described by Murad et al. [33] in a modified way. For every domain, the information in a publication was evaluated as good, moderate, or insufficient. The four domains were patient selection (Do the patients represent the whole experience of the investigator/center? Is the selection method unclear to the extent that other patients with similar presentation may not have been reported?), ascertainment (Were exposure and outcome adequately ascertained?), causality (Were other alternative causes that may explain the outcome ruled out?), and reporting (Are sufficient details given to allow other researchers to replicate the research or make inferences related to their own practice?).

### Data extraction from articles

From the available full-text articles, the following information was extracted: information on the comparison of mapping outcome of nTMS and DCS. For motor mapping, in most studies, the Euclidian distance was given between nTMS- and DCS-mapped cortical representations of muscle groups. For language studies, often sensitivity, specificity, positive predictive value (PPV), and negative predictive value (NPV) were used to evaluate nTMS mapping, by comparison of nTMS results with “gold standard” DCS mapping results. To obtain these data, most studies divide the cortical surface in many small cortical areas and calculate the (dys)congruence of the mapping results from the nTMS and DCS techniques for each of these areas. If available, the following information was also extracted: the number of patients in the study on which the comparison between nTMS and DCS was made (this did not always correspond to the total number of patients included in a study), the year of publication, and the disease type of the patient population. The type of TMS machine and other hardware used for the preoperative mapping procedure were noted. The nTMS mapping protocol and settings were recorded as completely as possible from the description in the article. A qualitative analysis was performed.

## Results

The initial database search yielded 2190 citations. After removal of duplicates and title and abstract screening, 62 citations remained for full-text reading. After the final step in the selection process, 35 articles were included in this review. Study design of the included articles and information regarding the domains described by Murad et al. were determined (Table 2). There were 26 publications with data about motor mapping and 10 publications with data about language mapping (one publication giving information on both motor and language mapping). No publications on the comparison of nTMS mapping and DCS mapping for other neurological functions were found. The publications appeared between 1997 and 2019.

**Table:**
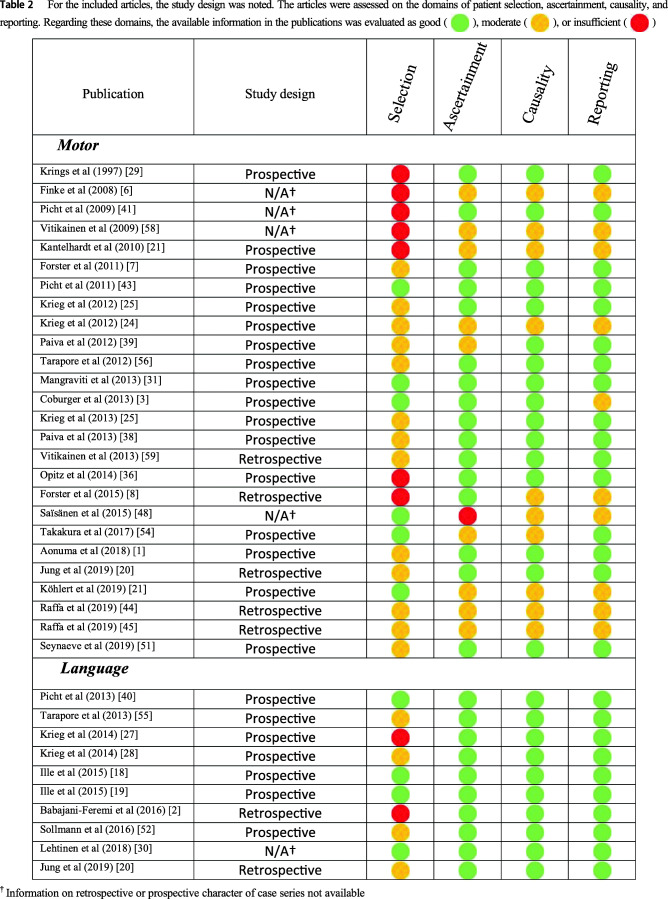

### nTMS for motor function

#### Patient population

Twenty-six articles with information about nTMS and DCS mapping of motor function were found, describing a total of 364 patients (Table 3) [1, 3, 6–8, 20–22, 24–26, 29, 31, 36, 38, 39, 41, 43–45, 48, 51, 54, 56, 58, 59]. The technique is used in tumor patients with different kinds of histopathology (high-grade/low-grade glioma, metastasis, meningioma, DNET, lymphoma, hemangiopericytoma, ganglioma, and cavernoma), in patients with vascular lesions, and in epilepsy patients. In all studies, patients tolerated nTMS mapping well. No adverse events were being mentioned.

**Table 3:** Articles describing the comparison of nTMS and DCS mapping of motor function

Author (year)	Number of patients	Pathology of patient population	TMS device/hardware	Information on TMS protocol	Distance nTMS and DCS	Sensitivity	Specificity	NPV	PPV	Other outcome measures	Conclusion
Krings et al. (1997) [29]	2	Tumors near the central sulcus	Magstim stimulator	Monophasic pulse < 1 ms. Stimulating 10% above RMT	n/a	n/a	n/a	n/a	n/a	TMS responses ≥ 75% of the maximum MEP were within 1 cm of regions where ECS elicited movements. Correlation coefficients of 0.57, 0.69, and 0.90 for the relationship between ECS and TMS sites for muscle groups. No TMS responses < 50% of maximum MEP were within 0.5 cm of DCS positive spots	Stereotactic TMS is feasible and can provide accurate non-invasive localization of cortical motor function. It may prove to be useful for presurgical planning
Finke et al. (2008) [6]	6	Tumors (WHO grades 1–4) neighboring the central region	MagPro stimulator with a Magstim coil and Polaris tracking system	10 pulses per spot with an interstimulus interval of 5 s	Three muscle groups mapped with robot-aided nTMS and DCS. The resulting maps agreed within 5 mm	n/a	n/a	n/a	n/a	n/a	Individual muscles or muscle groups can be detected reliably and with high accuracy in the motor cortex of patients with robotic nTMS. Displacement of brain regions due to tumor can be determined with robotic nTMS, as was confirmed by direct intraoperative stimulation
Picht et al. (2009) [42]	10	Rolandic tumors	MagPro stimulator and ACCISS II sensor–based navigation system	Stimulation at MT with increase in steps of 5% until compound muscle action potential of 100 μV	In 4 patients, the hotspots of nTMS and DCS were at identical location. In the other 6 patients, the mean distance was 3.4 mm (range 0–7 mm, SD 3.0 mm)	n/a	n/a	n/a	n/a	n/a	Navigated TMS functioned reliably and allowed detailed motor mapping. High congruence between the results of nTMS and the gold standard, DCS
Vitikainen et al. (2009) [58]	2	Epilepsy	Nexstim NBS system	Single-pulse nTMS. Stimulation at 105–110% of RMT	nTMS palm localization < 1 cm distance of ECS in the first patient	n/a	n/a	n/a	n/a	ECS-evoked movement of trunk and leg was not directly related to nTMS representations in patient 1. In patient 2, ECS elicited motor responses from the arm and hand, coinciding accurately with the locations defined by nTMS. ECS eliciting motor responses from the foot corresponded excellent to the preoperative nTMS localization	The results from MEG and nTMS localizations were consistent with the ECS results. These non-invasive methods can be added to the standard preoperative work-up
Kantelhardt et al. (2010) [21]	2	Tumors neighboring the precentral gyrus	MagPro stimulator with Adept Viper s850 robot for coil positioning and Polaris tracking system	Ten biphasic pulses per target with 5-s interstimulation interval. Stimulations at 40–45% of MSO	Patient 1: 26.3 mm AFTER resection Patient 2: < 5 mm BEFORE resection and 16.9 mm AFTER resection (in both cases, there was brainshift during surgery)	n/a	n/a	n/a	n/a	n/a	Robot-assisted image-guided TMS is a feasible and safe technique for non-invasive somatotopic mapping of the motor cortex. TMS may help in the planning of neurosurgical procedures
Forster et al. (2011) [7]	10	Lesions in or adjacent to the motor cortex	Nexstim NBS system	Mapping with stimulation at 110% of RMT	10.49 ± 5.67 mm (range 2.6–27.6 mm)	n/a	n/a	n/a	n/a	n/a	The study suggests that nTMS enables the visualization of eloquent cortex, not only in normal but also in distorted anatomy. It may complement the preoperative estimation of possible tumor removal
Picht et al. (2011) [43]	17	Rolandic tumors (glioma, metastasis, meningioma, lymphoma, cavernoma)	Nexstim NBS system	Stimulation at 110% of RMT and 0.25 Hz	Mean distance ± SEM between hotspots was 7.83 ± 1.18 mm for APB and 7.07 ± 0.88 mm for TA	n/a	n/a	n/a	n/a	n/a	Peritumoral mapping of the motor cortex by nTMS agreed well with the gold standard of DCS. Thus, nTMS is a reliable tool for preoperative mapping of motor function
Krieg et al. (2012) [25]	14	Lesions within or adjacent to the precentral gyrus (glioma, metastasis, DNET)	Nexstim NBS system with Polaris tracking system	Biphasic nTMS with stimulation at 110% of RMT, increasing to 130% for lower extremity	4.4 ± 3.4 mm (range 1.9–9.2 mm)	n/a	n/a	n/a	n/a	n/a	nTMS results highly correlate with intraoperative DCS and is possible in almost every case
Krieg et al. (2012) [24]	30	Tumors within the motor system (glioma, DNET, meningioma, AVM, metastasis)	Nexstim NBS system	n/a	4.5 ± 3.5 mm (range 1.9–9.2 mm)	n/a	n/a	n/a	n/a	n/a	nTMS is feasible in every patient, without discomfort and highly correlates with intraoperative DCS
Paiva et al. (2012) [39]	6	Low-grade glioma near the precentral gyrus	TMS device not mentioned; BrainSight neuronavigation system	Single-pulse TMS at 120% of RMT	Mean distance 4.16 ± 1.02 mm (range 2.56–5.27 mm)	n/a	n/a	n/a	n/a	The TMS and DES hotspots were located on the same gyrus in all cases	Preoperative mapping of the motor cortex with nTMS is a useful presurgical tool with good accuracy
Tarapore et al. (2012) [56]	24	Patients with tumors in proximity to the motor cortex (gliomas, radiotherapy effect)	Nexstim NBS system	Mapping at 110% of RMT. Peeling depth 23–28 mm	Median distance ± SEM 2.13 ± 0.29 mm	n/a	n/a	n/a	n/a	All patients who had negative DCS mapping also had negative TMS mapping	Maps of the motor system generated with TMS correlate well with DCS
Mangraviti et al. (2013) [31]	7	Lesion in eloquent motor area (gliomas, metastasis, cavernoma)	Nexstim NBS system	Mapping at 110 and 130% of RMT for upper and lower extremity, respectively. Peeling depth 20 mm. Maximum electric field 70 and 125 V/m for upper and lower extremity, respectively	Mean deviation/Euclidian distance 8.47 mm (CI 4.6 mm)	n/a	n/a	n/a	n/a	Separate distances for nTMS and DCS mapping for different muscle groups are given	nTMS data are in closer agreement with intraoperative DCS mapping than fMRI
Coburger et al. (2013) [3]	23	Lesion within or adjacent to the primary motor cortex (glioma, metastasis, ganglioma, hemangiopericytoma, meningioma, cavernoma, AVM)	Nexstim NBS system	Mapping performed using 110% of RMT	n/a	n/a	n/a	n/a	n/a	Grading system for TMS accuracy: 1. Distinct gyral localization 2. Projection over adjacent sulcus 3. Projection over adjacent gyrus 4. Inability to localize motor cortex. Mean score motor localization nTMS upper extremity 2.4 and lower extremity 2.1	nTMS has fewer restrictions than fMRI. The spatial resolution is more precise than fMRI. nTMS represents a highly valuable supplement for preoperative functional planning in the clinical routine
Krieg et al. (2013) [26]	31	Tumors located in or near the precentral gyrus or the corticospinal tract (8 recurrent gliomas, 23 newly diagnosed tumors)	Nexstim NBS system and Polaris tracking system	Upper extremity mapping at 110% of MT. Lower extremity mapping at up to 130% of MT	Recurrent tumors:● Mean distance 6.2 ± 6.0 mm ● Median distance 3.2 mm (range 0.0–18.2 mm) Newly diagnosed tumors:● Mean distance 5.7 ± 4.6 mm ● Median distance 4.5 mm (range 0.0–22.5 mm)	n/a	n/a	n/a	n/a	n/a	nTMS is as accurate in recurrent gliomas as it is prior to the first operation
Paiva et al. (2013) [38]	3	Cavernous angiomas	MagPro stimulator and BrainSight 2 neuronavigation device	Single-pulse stimulation at 120% of MT	Distance between hotspots 4.7 mm	n/a	n/a	n/a	n/a	n/a	Authors report for the first time TMS mapping in patients with cavernous angioma. The method appears to be a useful preoperative tool
Vitikainen et al. (2013) [59]	13	Focal epilepsy	Nexstim NBS system	Stimulation with monophasic pulse, only biphasic pulse if monopulse was not conclusive. Mapping with 105–110% of RMT	● Hand muscle: 11 ± 4 mm (7–17 mm) ● Arm muscle: 16 ± 7 mm (6–23 mm)	n/a	n/a	n/a	n/a	n/a	Concordance between nTMS and ECS in epilepsy patients is in the similar range as reported for tumor patients
Opitz et al. (2014) [36]	6	Tumors in vicinity of the motor cortex (metastasis, glioblastoma)	MagPro stimulator with Visor2 neuronavigation system	Single-pulse stimulation at 120% of RMT. 10 pulses per position with 4-s interpulse interval and 400-ms jitter	Euclidian distance Realistic model: 6.3 ± 0.7 mm Spherical model: 8.9 ± 1.7 mm	n/a	n/a	n/a	n/a	Stimulation areas in TMS and DES show an overlap of up to 80% (for realistic TMS model)	Compared with spherical models, realistic models make a more specific prediction of TMS target areas which are in better accordance with the DES results
Forster et al. (2015) [8]	6	Perirolandic tumors (glioma, metastasis)	Nexstim NBS system	Single-pulse stimulation with mapping at 110% of RMT	n/a	n/a	n/a	n/a	n/a	Localization of the hand representation area by nTMS and DCS was consistent in all patients	Findings from nTMS and intraoperative direct cortical mapping of the hand motor cortex were congruent in all cases
Saïsänen et al. (2015) [48]	6	Tumor near the eloquent hand and/or facial motor areas (glioma, cavernoma)	Nexstim NBS system	Mapping with single-pulse stimulation at 105/110% of facial MT. Peeling depth 16.7–27.0 mm	n/a	n/a	n/a	n/a	n/a	DCS and TMS mapping was congruent in 3 out of 6 patients	nTMS is useful for cortical mapping of facial muscles. The MT of facial muscles is higher than that of small hand muscles
Takakura et al. (2017) [54]	13	Intraparenchymal brain neoplasms located within or adjacent to the motor eloquent area	Rapid square Magstim stimulator with BrainSight navigation system and Polaris tracking system	Mapping with 0.25-Hz stimulation at 110% of RMT or 100–110% of AMT with single biphasic pulse and 4-s interval	n/a	n/a	n/a	n/a	n/a	MEP inducibility by presurgical nTMS and response to DES were significantly associated (*p* < 0.05)	Hotspots for ABP identified by nTMS were concordant with DES-positive sites
Aonuma et al. (2018) [1]	4	Gliomas within or close to motor eloquent area	Rapid square Magstim stimulator with BrainSight TMS navigation system	Mapping at 110% of RMT with 0.25-Hz stimulation	7.78 ± 1.99 (SE) mm	n/a	n/a	n/a	n/a	n/a	The study presents a new method to estimate the hotspot by nTMS and shows a better result than the hotspot predicted by the navigation system
Jung et al. (2019) [20]	11	Gliomas, metastasis, epidermoid cyst, and cavernoma	Nexstim NBS system	The TMS hotspot was demarcated as the stimulation position eliciting the strongest MEP at 105% of RMT	Mean distance 3.50 ± 0.66 mm for APB localization	n/a	n/a	n/a	n/a	In 11 patients, nTMS and DES hotspots were located on the same gyrus	nTMS is a safe, non-invasive adjunctive tool for preoperative mapping of space-occupying lesions in eloquent areas
Kohlert et al. (2019) [22]	30	Tumors in or near by the primary motor cortex region (8 glioblastoma, 4 meningioma, 14 metastasis, 1 radiotherapy necrosis, 3 lymphoma)	MagPro stimulator with LOCALITE stereotactic positioning system	Stimulation with single pulses. Stimulation varied from 51 to 100% of maximum stimulation strength	n/a	n/a	n/a	n/a	n/a	The localization of the primary motor cortex by TMS corresponded well with intraoperative direct motor stimulation findings in all cases. The semi-quantitative result was verified by a screen shot with the navigational system	The nTMS allows safe and reliable preoperative mapping of the primary motor cortex, with good correspondence to DCS
Raffa et al. (2019) [44]	35	Convexity, parasagittal and falxmeningiomas near the rolandic area	Nexstim NBS system	Calculation of the RMT. The M1 mapping was performed as previously reported (e.g., Picht et al.)	n/a	93.5%	100%	66.6%	100%	Intraoperative mapping and nTMS mapping were concordant in all but 2 cases (5.7%). The accuracy of the nTMS-based mapping compared with intraoperative mapping was 94.2%	nTMS-based mapping of the motor pathway can be considered a useful presurgical tool for treatment of rolandic meningiomas
Raffa et al. (2019) [45]	41	High-grade gliomas in or close to (< 11 mm) the motor area	Nexstim NBS system	Single-pulse stimulation at 120% of RMT	n/a	100%^i^	80%^i^	100%^i^	89.66%^i^	nTMS-based reconstruction of the entire motor pathway (M1 and CST) had an accuracy of 92.68% compared with intraoperative mapping	The multimodal approach of sodium fluorescein–guided resection with nTMS-based preoperative reconstruction of the motor pathway could facilitate achievement of maximal safe resection
Seynaeve et al. (2019) [51]	12	Perirolandic tumors	Magstim stimulator. BrainSight navigation system	EMG measurement of APB and TA. Stimulation at 110% of the RMT and kept constant during the procedure	Euclidian distance 11 ± 1.5 (SD) mm	Range between 38.3 and 83.2% for different TMS analysis methods	Range between 66.7 and 88.7% for different TMS analysis methods	n/a	n/a	Accuracy 83–86% for locating the motor cortex	The method with which TMS mapping data are analyzed clearly affects the predicted area of the primary motor cortex representation. Realistic electric field–based modeling improves delineation of the motor cortex representation

*RMT* resting motor threshold, *n/a* not available, *MEP* motor evoked potential, *ECS* electrical cortical stimulation, *MT* motor threshold, *NBS* navigated brain stimulation, *MSO* maximum stimulator output, *APB* abductor pollicis brevis, *TA* tibialis anterior, *DES* direct electrical stimulation, *AMT* active motor threshold, *CST* corticospinal tract, *EMG* electromyography

^i^For nTMS-based reconstruction of the entire motor pathway (M1 and CST)

#### nTMS motor mapping protocols

Different nTMS protocols were used for motor mapping. In 22 articles, the (resting) motor threshold (R)MT was determined. Two articles only mention the percentage of maximum stimulator output (MSO) that was applied [21, 22]. Another article also used the active motor threshold (AMT) in some patients to determine the stimulator output setting [54]. In the articles where (R)MT was obtained, the stimulation intensity varied from 105 to 130% of (R)MT for mapping of motor function.

#### Muscle groups mapped with nTMS

In the included studies, hand, arm, leg, and facial muscles are mapped with nTMS. Hand muscles are most often mapped (in all 26 available articles), followed by leg muscles (in 14 out of 26 articles).

#### Comparison of nTMS and DCS motor mapping

Eighteen articles describe the distance between nTMS-mapped functional points and DCS-mapped functional points as an outcome measure. Average/median (Euclidian) distances of 2.13–16 mm are reported. The data in the publication of Kantelhardt et al. [21] were not taken into consideration here, because the authors give a distance between nTMS mapping and DCS mapping after removal of the tumor. In this situation, most likely brainshift will have occurred. Fourteen articles describe an accuracy of < 10 mm for nTMS motor mapping compared with DCS mapping. Most authors conclude that nTMS motor mapping is reliable compared with DCS mapping.

#### nTMS devices for motor mapping

For motor mapping, 15 out of 26 articles used a Nexstim navigated brain stimulation (NBS) system (Helsinki, Finland). Two other manufacturers of TMS devices, Magstim (Whitland, UK) and MagVenture (Farum, Denmark), could be identified in the included publications.

### nTMS for language function

#### Patient population

Ten publications give information about language mapping with nTMS and DCS, describing in total 188 patients (Table 4) [2, 18–20, 27, 28, 30, 40, 52, 55]. In one of their publications, Ille et al. mention that some patient data have been used in previous studies [19]. The use of patient data in multiple publications could potentially make the total number of patients described in the literature, regarding the comparison of nTMS language mapping and DCS language mapping, lower than the number mentioned here*.* In the included studies in this review, language function in tumor patients and epilepsy patients was mapped. No adverse events are mentioned in the studies. The nTMS language mapping was also well tolerated in pediatric patients according to Lehtinen et al. [30]. In the study, 14 pediatric and adolescent patients were included, with an age ranging from 9 to 18 years.

**Table 4:** Articles describing the comparison of nTMS and DCS mapping of language function

Author (year)	Number of patients	Pathology of patient population	TMS device/hardware	Information on TMS protocol	Distance nTMS and DCS	Sensitivity	Specificity	NPV	PPV	Other outcome measure	Conclusion
Picht et al. (2013) [40]	20	Tumors close to left-sided language-eloquent region	Nexstim NBS system	NexSpeech module with object naming task. 10–20 TMS bursts, 5–10 Hz, 80–120% RMT. Interpicture interval 2–4 s	n/a	● Overall sensitivity 90.2% ● Broca area sensitivity 100%	● Overall specificity 23.8% ● Broca area sensitivity 13%	● Overall NPV 83.9% ● Broca area NPV 100%	● Overall PPV 35.6% ● Broca area PPV 56.5%	n/a	Good overall correlation between repetitive nTMS and DCS was observed, particularly with regard to negatively mapped regions
Tarapore et al. (2013) [55]	12	Brain tumors in cortical language areas	Nexstim NBS System	Language mapping at 110% of RMT, train of 10 pulses at 5 Hz for 2 s. Interstimulus interval 4 s. Image visibility 3 s. TMS pulse at stimulus onset	n/a	90%	98%	99%	69%	n/a	Maps of language function generated with nTMS correlate well with those generated by DCS
Krieg et al. (2014) [27]	3	Glioma (primary and repeated awake resection)	Nexstim NBS system	Object naming task. rTMS with 5 to 7 pulses, 5 to 7 Hz at 100–110% RMT	n/a	10% for ≥ 25% error rate	89% for ≥ 25% error rate	82% for ≥ 25% error rate	17% for ≥ 25% error rate	ROC characteristics table for different error rates available	rTMS is able to partially detect language-negative regions prior to surgery or during follow-up
Krieg et al. (2014) [28]	32	Left-sided perisylvian tumors	Nexstim NBS system	NexSpeech module. 20 patients had TMS pulse train onset at 300 ms after picture presentation vs. 12 patients with pulse train onset at 0 ms after picture presentation	n/a	Broca area ● 0 ms^i^ 100% ● 300 ms^i^ 100% Posterior language regions ● 0 ms^i^ 75% ● 300 ms^i^ 70%	Broca area ● 0 ms^i^ 67% ● 300 ms^i^ 28% Posterior language regions ● 0 ms^i^ 92% ● 300 ms^i^ 20%	Broca area ● 0 ms^i^ 100% ● 300 ms^i^ 100% Posterior language regions ● 0 ms^i^ 92% ● 300 ms^i^ 57%	Broca area ●0ms^i^ 55% ●300ms^i^ 54% Posterior language regions ● 0 ms^i^ 75% ● 300 ms^i^ 30%	n/a	The study demonstrates that rTMS stimulation onset at 0 ms, coincident with picture presentation, improves the accuracy of preoperative language mapping
Ille et al. (2015) [18]	27	Left-sided perisylvian lesions	Nexstim NBS system	NexSpeech module. Stimulation with 80–120% of RMT, 5–7 pulses at 5–7 Hz. Interpicture interval 2500 ms, picture display time 700 ms, picture-to-trigger interval 0 ms (5 patients) and 300 ms (22 patients)	n/a	97%^ii^	15%^ii^	91%^ii^	34%^ii^	Separate analysis for anterior and posterior regions is performed	The study shows that rTMS language mapping is less affected by presence of a brain lesion than fMRI
Ille et al. (2015) [19]	35	Left-sided perisylvian lesions	Nexstim NBS system	NexSpeech module. Stimulation with 80–120% of RMT, 5–7 Hz, interpicture interval 2.5 s, picture display time 700 ms, picture-to-trigger interval 0 ms (10 patients) and 300 ms (25 patients)	n/a	67%^iii^	49%^iii^	79%^iii^	34%^iii^	Outcomes for different ERTs and picture-to-trigger intervals are given	rTMS is most reliable for language mapping when ERTs of 15%, 20%, 25% or the 2-out-of-3 rule are used and a picture-to-trigger interval of 0 ms
Babajani-Feremi et al. (2016) [2]	9	Epilepsy (no brain lesion in vicinity of language cortex)	Nexstim NBS system and Polaris tracking system	NexSpeech module. Object naming task. Stimulation with 5 pulses at 5 Hz. Interpicture interval 2.5–4 s. Interval TMS and picture presentation 0–200 ms. Intensity adjusted to E-field delivery 80–100 V/m at a depth of 20–25 mm	Euclidian distance 8.7 ± 3.8 mm	67%	66%	95%	24%	Area under the ROC curve 0.68	There is considerable concordance between cortical stimulation mapping, high gamma electrocorticography, fMRI, and TMS for language mapping. They are valuable tools for presurgical language mapping
Sollmann et al. (2016) [52]	20	Left-sided perisylvian brain lesions	Nexstim NBS system	NexSpeech module. According to protocol described in recent investigations	n/a	● 92.7% (without ERT) ● 68.3% (2-out-of-3 rule) ● 51.2% (ERT ≥ 20%)	● 13.3% (without ERT) ● 50.8% (2-out-of-3 rule) ● 64.2% (ERT ≥ 20%)	● 84.2% (without ERT) ● 82.4% (2-out-of-3 rule) ● 79.4% (ERT ≥ 20%)	● 26.8% (without ERT) ● 32.2% (2-out-of-3 rule) ● 32.8% (ERT ≥ 20%)	n/a	The additional use of rTMS-based diffusion tensor imaging fiber tracking to rTMS did not improve the identification of DCS-positive language areas
Lehtinen et al. (2018) [30]	20	Epilepsy (pediatric and adult patients)	Nexstim NBS system	NexSpeech module. Object naming task. Stimulation at 76–100% of RMT with 5–7 pulses of 5–7 Hz. Picture display time 0.7–1 s, interpicture interval 2.5–5 s, picture-to-stimulation interval 0–500 ms	n/a	68%	76%	95%	27%	Separate analysis for anterior and posterior regions is performed	nTMS language mapping is clinically useful and safe in epilepsy surgery patients. Obtained sensitivity, specificity, and predictive values are generally in line with previous results
Jung et al. (2019) [20]	10	Glioma, metastasis, epidermoid cyst, and cavernoma	Nexstim NBS system	NexSpeech module. Stimulation with 5 pulses at 5 Hz. 700-ms picture presentation time, 2500–3000-ms picture interval, 0-ms delay between stimulation and picture onset	n/a	63.2%	66.7%	74.1%	54.5%	n/a	Preoperative language mapping with nTMS confirmed a high NPV and specificity

*ERT* error rate threshold

^i^Interval between picture presentation and rTMS stimulation onset

^ii^Overall results all mapped regions

^iii^Here, results of the 2-out-of-3 rule are given

#### nTMS language mapping protocols

For language mapping, repetitive TMS (rTMS) is used to suppress part of the (sub)cortical network responsible for the production of language. All studies use the NexSpeech module of the Nexstim NBS system, in which an object naming task has to be performed. Stimulation was done at 76–120% of resting motor threshold (RMT). In the rTMS mapping protocols, between 5 and 20 TMS bursts were given, with a frequency ranging from 5 to 10 Hz. Other variables in the stimulation protocols were duration of picture display time (range 700 ms–3 s), interpicture interval (range 2–5 s), and picture-to-stimulation interval (range 0 s–500 ms).

#### DCS language mapping protocols

The DCS language mapping was conducted using the “Penfield technique” in eight studies, with a stimulation frequency of 40–60 Hz during 4 s. In six studies, intraoperative electrocorticography (ECoG) was applied to detect epileptic activity and afterdischarges after stimulation. In the two studies on epilepsy patients, a subdural grid electrode was used for DCS extraoperative language mapping.

#### Type of language error elicited by stimulation

All articles mention in their study protocols the type of language errors that are registered during the nTMS mapping procedure (speech arrest, performance error, hesitation, neologism, semantic paraphasia, phonologic paraphasia, circumlocution, anomia). Only Sollmann et al. analyzed the correlation between the type of language error that could be evoked with nTMS mapping and the type of error found with DCS mapping [52]. Lehtinen et al. give information about the percentage of true positive nTMS mapped types of language errors in relation to the DCS mapping outcome, which ranged between 14 and 76% [30].

#### Comparison of nTMS and DCS language mapping

Eight publications use a cortical parcellation system (CPS) for language mapping. In this model, the hemisphere is divided into 37 anatomical regions. The two other studies use the Montreal Neurological Institute (MNI) coordinate system. The comparison between language positive and negative nTMS and DCS points makes calculation of sensitivity, specificity, NPV, and PPV possible. Sensitivity ranged from 10 to 100% and specificity ranged from 13.3 to 98%. NPV and PPV ranged from 57 to 100% and 17–75%, respectively. Cut-off values, regarding when a cortical region is considered positive or negative for language function, strongly influenced these outcomes. The negative mapped areas clearly had the highest predictive value. One study mentioned distance between nTMS- and DCS-mapped points as an outcome measure. Babajani-Feremi et al. [2] described a Euclidian distance of 8.7 mm between nTMS- and DCS-mapped localizations. In four articles, a separate analysis for the posterior and anterior language areas and nTMS mapping accuracy was performed. The anterior (Broca’s) language areas had the most reliable nTMS mapping results. Most articles conclude that nTMS language mapping is clinically useful, especially in regard to negatively mapped regions.

#### nTMS devices for language mapping

All nTMS language mapping was done with the Nexstim equipment and software. There was no diversity in manufacturer of the device and language testing software.

## Discussion

nTMS is a relatively new, promising mapping technique for cortical function localization. This article provides an overview of the available literature on the comparison of nTMS with DCS mapping in the neurosurgical practice. At the moment, comparative data are only available for motor and language mapping. For other modalities (e.g., arithmetic function/calculation, neglect/spatial function, visual field aspects), only non-comparative data in healthy subjects and sometimes patients are available [11, 12, 14, 32, 49]. This renders multiple unresolved questions for future research. Comparing the results of nTMS and DCS mapping for other modalities can help cross-validate the results of the relatively new motor and language literature. The number of centers publishing their data on nTMS mapping is growing but still limited, with some centers being the predominant publishers/collaborators. Especially the Munich and Berlin neurocenters have a broad experience with the nTMS mapping technique and are responsible for 31% of the publications included in this systematic review.

### nTMS motor mapping

The largest body of evidence is available for nTMS motor mapping. The technique has proven to be reliable on a scale of millimeters compared with DCS in a large number of studies (Table 3). Hand motor function is the most frequently mapped cortical area. However, other muscles can be mapped with the nTMS technique as well. There is an extensive body of literature on nTMS motor mapping, forming a solid base for its application in clinical practice. Although there is excellent agreement between preoperative nTMS motor mapping and DCS motor mapping, intraoperative monitoring of the pyramidal tract and SCS are still indispensable, to secure integrity of the entire motor pathway.

### nTMS language mapping

Language mapping with nTMS has also extensively been described in the literature, albeit to a lesser extent than motor mapping. The technique of language mapping is more complex, because language is the result of a network function, which is more difficult to localize and map than a circumscriptive motor area in the precentral gyrus [47]. In the studies on nTMS language mapping, a notable large variability in sensitivity, specificity, NPV, and PPV was observed. The different values are highly dependent on the criteria that are used to determine if a cortical area is considered positive or negative for language function. In DCS mapping during awake surgery, the 2-out-of-3 rule is commonly applied, to decide if a specific area is language eloquent or not. This rule implicates that a cortical area is stimulated three times. The specific localization will be regarded eloquent for the tested neurological function, if a performance error can be provoked at least two times due to stimulation. If the amount of positive stimulations is less than 2-out-of-3, then the area is regarded non-eloquent for the tested neurological function. The nTMS mapping error rate, which is used as a cut-off value for positive or negative language function localization, greatly influences sensitivity, specificity, NPV, and PPV [19]. For the moment, there are no guidelines available, advising on which error rates should be used as a cut-off in deciding on eloquence versus non-eloquence in nTMS language mapping.

Also, methodological differences influence accuracy results of the included studies, as has been pointed out by Tarapore et al. [54]. For example, their use of a more dense grid to separate different mapped brain regions than other groups and a standardized data normalization algorithm can explain the relatively high sensitivity, specificity, and NPV in their publication. Furthermore, the methodological definition of nTMS/DCS concordance versus nTMS/DCS non-concordance influences the accuracy outcomes.

False-negative nTMS-mapped regions are, of course, a major concern. However, the total number of false-negative nTMS-mapped areas is low in all included studies. The false-negative areas occurred predominantly in the posterior language areas.

Most authors conclude that nTMS language mapping is a very useful preoperative tool, but the technique cannot replace DCS during awake surgery and it should be used as an adjunct to awake intraoperative testing. There is no supportive literature for resection of language-eloquent lesions based on nTMS functional data alone. Only in patients in which an awake procedure is not feasible (e.g., due to a psychiatric condition or in young children), it can be considered to perform a resection without awake testing based on nTMS mapping results (combined with DTI-FT) as has been described by Ille et al. [17]. In their case series of four patients, who did not qualify for awake surgery and had a nTMS-based resection under general anesthesia, no new neurological deficits occurred. However, one patient underwent a second resection several days after the primary procedure to achieve a complete resection. The authors advocate that nTMS-based resection can only form a “rescue strategy” for patients who do not qualify for awake surgery.

### nTMS mapping protocols

Regarding nTMS mapping protocols, our findings show that differences in mapping protocols exist. In recent literature, this is especially true for language mapping protocols. For example, the publication from Krieg et al. shows that the timing of nTMS pulse onset after picture presentation influences the nTMS mapping result [28]. Also, differences in the number of TMS bursts and time intervals (interpicture interval, picture-display-time) exist. This opens possibilities for future directions/perspectives. A recent consensus meeting about the protocol for motor and language mapping, however, has helped to overcome major diversity in current practice [23]. During this meeting, participating experts agreed that there is enough supportive evidence for the use of nTMS motor mapping in routine clinical practice. Details on the nTMS motor mapping protocol are given in the supplementary material of the meeting report [23]. In the opinion of this consensus group, nTMS language mapping should be performed in the framework of clinical studies. In the meeting report, a nTMS language mapping protocol is proposed and the parameters are appointed that should be taken into consideration when performing nTMS language mapping. It is stated, however, that further refinement of this protocol is necessary [23]. Optimization of nTMS mapping protocols should be achieved, primarily, by testing different settings in healthy subjects.

Furthermore, there is a need for standardization regarding the interpretation of nTMS responses. Especially cut-off values when a stimulated area is considered positive or negative should become more clearly established in future protocols. Besides, in most nTMS language mapping sessions, only an object naming task was performed. There are no data on nTMS mapping test batteries containing, for example, verb generation, reading, and writing, and the comparison with DCS mapping results of those functions. Also, data regarding the correlation between type of language errors (e.g., speech arrest, anomia, phonemic paraphasia, semantic paraphasia, hesitation) in nTMS and DCS mapping procedures are scarce.

In DCS mapping, ECoG is frequently used intraoperatively to be informed about epileptic activity and afterdischarges following cortical stimulation, which can form an alternative explanation for language errors. The addition of electroencephalography (EEG) to nTMS mapping is not applied in most protocols. The combination of both techniques could possibly make the interpretation of stimulation results in nTMS language mapping more accurate. Although during nTMS mapping hardly any epileptic seizures have been encountered according to the literature, it could be interesting to know if a nTMS-provoked speech disturbance is a very focal effect, or that the stimulation maybe did cause a more widespread disturbance/epileptiform activity in patients than is currently believed.

### nTMS mapping devices

The diversity in TMS hardware/devices is limited. Predominantly Nexstim NBS (Helsinki, Finland) machines were used. In total, 24 out of 35 publications use the Nexstim equipment. Two other manufacturers of nTMS devices for mapping of neurological function could be identified in the included articles in this review, which are Magstim (Whitland, UK) and MagVenture (Farum, Denmark).

### nTMS mapping and fMRI function localization

nTMS mapping has been compared with fMRI mapping in several studies as well. For motor function localization, there is support that nTMS mapping is more accurate than fMRI [3, 7, 30] and, in addition, the distance between nTMS- and DCS-mapped functional regions is smaller than the distance between nTMS- and fMRI-mapped functional regions [23–25]. Regarding mapping of language function, nTMS mapping is more sensitive, but less specific than fMRI [2, 18].

### nTMS mapping for other purposes than preoperative cortical function localization

Although initially during its development in the neurosurgical practice, the focus was on nTMS being a preoperative mapping tool, many new applications have been described recently. There is literature describing nTMS as a tool to investigate plasticity and shift of neurological function localization over time in patients suffering from different neurological conditions [15]. With this, nTMS becomes an instrument to judge the possibility of secondary craniotomies after the primary procedure because, due to the plasticity and shift of neurological function, new opportunities for safe resections might become possible in the course of the disease, which were not possible during the primary procedure due to eloquence. Also, nTMS data are successfully used as seeding point for diffusion tensor imaging (DTI) fiber tracking of white matter tracts [4, 9, 34, 35, 60]. There is literature describing nTMS mapping as a helpful tool in the planning of radiosurgery targets in eloquent brain regions [5, 42, 50, 57]. Last but not least, nTMS is used as a method to determine eloquence and thus classification of arteriovenous malformations and, depending on this classification, the treatment of those lesions [16]. All these purposes are useful in clinical practice. However, it must be emphasized that the accuracy of the nTMS technique remains the pivotal mainstay for all aforementioned purposes. The current review gives an overview of the available data concerning this topic.

### Study limitations

The data in this systematic review were not deemed suitable for meta-analysis because of the diversity in outcome measures and because it cannot be excluded that some patient data are used in multiple studies. The included studies have a prospective or retrospective character. Hence, in a number of studies, the data were collected primarily for clinical, not comparative, purposes. This review compares nTMS mapping with DCS mapping; in most included articles, this concerned intraoperative DCS mapping, but in the articles on epilepsy patients, an operatively placed subdural grid was used for DCS extraoperative language mapping. Both DCS techniques are not fully comparable. In this systematic review, the effect of nTMS mapping on treatment outcome was not evaluated.

## Conclusion

nTMS mapping is a relatively new mapping technique for cortical function localization and can be a helpful and informative preoperative diagnostic tool. The largest body of evidence is available for nTMS motor mapping, in which the accuracy compared to DCS mapping is good. Concerning nTMS language mapping, there is more variability in accuracy results. The technique cannot replace intraoperative language mapping and should be used as an adjunct. The NPV and sensitivity of nTMS language mapping seem to be the most reliable, when nTMS is compared with DCS, especially in the anterior language areas. For now, only for nTMS motor and language mapping, comparative data with DCS are available. For other neurological functions, no comparative literature between both techniques is available yet. Further work should emphasize on the validation of nTMS mapping for other neurological functions, as well as other language tasks.

## Electronic supplementary material

Supplement 1 Search strings used in the three different electronic databases (DOCX 13 kb)
