# Supplementary material for: Comparing navigated transcranial magnetic stimulation mapping and “gold standard” direct cortical stimulation mapping in neurosurgery: a systematic review
Source: Neurosurg Rev. 2020 Oct 3;44(4):1903–20. doi: 10.1007/s10143-020-01397-x (PMC8338816; doi:10.1007/s10143-020-01397-x)
Supplement: Supplementary file 1 — Search strings used in the three different electronic databases (DOCX 13 kb) [file 10143_2020_1397_MOESM1_ESM.docx]

| **PubMed**  ((navigated transcranial magnetic stimulation OR navigated TMS)  **OR**  (("Transcranial Magnetic Stimulation"[Mesh] OR transcranial magnetic stimulation*[tiab] OR TMS[tiab] OR rTMS[tiab])  **AND**  (((intraoperat*[tiab] OR intra-operat*[tiab] OR during surg*[tiab] OR (awake[tiab] AND surgery[tiab]) OR intracranial[tiab]) *AND (*mapping[tiab] OR cortical stimulat*[tiab] OR subcortical stimulat*[tiab])) OR  direct cortical stimulat*[tiab] OR direct electrical stimulat*[tiab] OR  cortical stimulation mapping[tiab] OR intraoperative stimulat*[tiab] OR intra-operative stimulat*[tiab] OR direct stimulation[tiab] OR ((direct[tiab] NOT (“direct current”[tiab] OR tdcs[tiab])) *AND* (cortical stimulat*[tiab] OR electrical stimulat*[tiab])) OR dcs[tiab]))) **NOT**  ("Animals"[Mesh] NOT "Humans"[Mesh])  **EMBASE**  ((‘navigated transcranial magnetic stimulation’ OR ‘navigated TMS’) **OR**  (('transcranial magnetic stimulation'/exp OR (‘transcranial magnetic stimulation*’ OR TMS OR rTMS):ab,ti)  **AND** (((intraoperat* OR ‘intra-operat*’ OR ‘during surg*’ OR (awake AND surgery) OR intracranial) AND (mapping OR ‘cortical stimulat*’ OR ‘subcortical stimulat*’)) OR  ‘direct cortical stimulat*’ OR dcs OR ‘direct electrical stimulat*’ OR  ‘cortical stimulation mapping’ OR ‘intraoperative stimulation’ OR ‘intra-operative stimulation’ OR ‘direct stimulation’ OR  ((direct NOT (‘direct current’ OR tdcs)) AND (‘cortical stimulat*’ OR ‘electrical stimulat*’)) OR dcs):ab,ti)) **NOT**  (('animal'/exp OR 'nonhuman'/exp) NOT 'human'/exp) **NOT**  'conference abstract'/it  **Web of Science**  TS= (“navigated transcranial magnetic stimulation” OR “navigated TMS”)  OR  (TS=((“transcranial magnetic stimulation” OR TMS OR rTMS)  AND  (intraoperat* OR intra-operat* OR surger* OR surgic* OR intracranial OR “direct stimulation” OR “direct cortical” OR dcs)  AND  (mapping OR stimulat* OR dcs))) |
| --- |
